# The effect of tumor volume and its change on survival in stage III non-small cell lung cancer treated with definitive concurrent chemoradiotherapy

**DOI:** 10.1186/s13014-014-0283-6

**Published:** 2014-12-13

**Authors:** Tae Ryool Koo, Sung Ho Moon, Yu Jin Lim, Ja Young Kim, Yeonjoo Kim, Tae Hyun Kim, Kwan Ho Cho, Ji-Youn Han, Young Joo Lee, Tak Yun, Heung Tae Kim, Jin Soo Lee

**Affiliations:** Department of Radiation Oncology, Seoul National University College of Medicine, Seoul, Republic of Korea; Proton Therapy Center, Research Institute and Hospital, National Cancer Center, 323 Ilsan-ro, Ilsandong-gu, Goyang-si, Gyeonggi-do Korea; Center for Lung Cancer, Research Institute and Hospital, National Cancer Center, 323 Ilsan-ro, Ilsandong-gu, Goyang-si, Gyeonggi-do Korea

**Keywords:** Concurrent chemoradiotherapy, Gross tumor volume, Locoregional control, Non-small-cell lung cancer, Survival

## Abstract

**Background:**

To investigate a prognostic role of gross tumor volume (GTV) changes on survival outcomes following concurrent chemoradiotherapy (CCRT) in stage III non-small-cell lung cancer (NSCLC) patients.

**Methods:**

We enrolled 191 patients with stage III NSCLC from 2001 to 2009 undergoing definitive CCRT. The GTV of 157 patients was delineated at the planning CT prior to CCRT and with a follow-up CT 1 month after CCRT. We assessed the volumetric parameters of pre-treatment GTV (GTV_pre_) post-treatment GTV (GTV_post_), and volume reduction ratio of GTV (VRR). The primary endpoint was overall survival (OS) and secondary endpoints were progression-free survival (PFS) and locoregional progression-free survival (LRPFS). The best cut-off value was defined as that which exhibited the maximum difference between the two groups.

**Results:**

The median follow-up duration was 52.7 months in surviving patients. Median survival, 3-year OS, PFS and LRPFS rates were 25.5 months, 36.4%, 23.0%, and 45.0%, respectively. The selected cut-off values were 50 cm^3^ for GTV_pre_, 20 cm^3^ for GTV_post_, and 50% for VRR. The smaller GTV_pre_ and GTV_post_ values were associated with better OS (*p <* 0.001 and *p =* 0.015) and PFS (*p =* 0.001 and *p =* 0.004), respectively, upon univariate analysis. The higher VRR of > 50% was associated with a trend toward poorer OS (*p =* 0.004) and PFS (*p =* 0.054). Upon multivariate analysis, smaller GTV_pre_ indicated significantly improved OS (*p* = 0.001), PFS (*p =* 0.013) and LRPFS (*p* = 0.002), while smaller GTV_post_ was marginally significant for PFS (*p =* 0.086). Higher VRR was associated with a trend toward poorer OS (*p* = 0.075).

**Conclusions:**

In patients with stage III NSCLC undergoing definitive CCRT, GTV_pre_ was an independent prognostic factor of survival. Notably, improved outcome was not correlated with higher VRR after short-term follow-up with CT alone.

**Electronic supplementary material:**

The online version of this article (doi:10.1186/s13014-014-0283-6) contains supplementary material, which is available to authorized users.

## Background

Definitive radiotherapy with concurrent chemotherapy (CCRT) has been the mainstay of treatment for unresectable or medically inoperable stage III non-small cell lung cancer (NSCLC), and offers a greater chance of survival compared with sequential chemoradiotherapy [1,2]. The American Joint Commission on Cancer (AJCC) TNM staging system is widely used to estimate the prognosis of patients treated with definitive radiotherapy (RT) for stage III NSCLC but is less informative for radiation oncologists [3-5]. The AJCC TNM staging system includes detailed size criteria in the staging of NSCLC since the size of primary tumor itself, as well as the anatomic location and invasiveness of the primary lesion, is consistently related to prognosis [4]. In patients with stage III NSCLC treated with CCRT, the response rate may reflect sensitivity to both RT and chemotherapy or tumor kinetics. However, it is not known whether the response rate or tumor volume changes after CCRT are associated with prognosis.

While a prognostic role for tumor volume in survival of patients with definitive RT has been reported [6-12], none of these studies investigated the effect of residual tumor volume or tumor volume change during treatment on the treatment outcome. In this retrospective study, we performed volumetric survival analysis to investigate the prognostic role of gross tumor volume (GTV) before and after treatment on survival outcomes in stage III NSCLC patients treated with CCRT.

## Methods

A total of 191 patients with stage III NSCLC underwent definitive CCRT from December 2001–January 2009 in our institution. A diagnosis of NSCLC was histologically confirmed and all patients re-staged with the 7^th^ AJCC TNM staging system [13]. All patients underwent pre-treatment imaging work up, including chest radiographs and computed tomography (CT). For the purpose of our study, 22 patients were excluded due to loss of follow-up immediately after completion of CCRT, incomplete RT of < 50 Gy, or planned surgery following treatment. Additionally, 12 patients whose initial tumor volume data was not available to be reviewed due to technical errors during image registration were excluded. Patient characteristics are listed in Table 1.

**Table 1:** **Patients’ characteristics**

**Characteristics**		***n***	**(%)**
Age (y)	Median (range)	63	(range, 27–81)
Sex	Male	137	(87%)
	Female	20	(13%)
Performance	ECOG 0	39	(25%)
	ECOG 1	111	(71%)
	ECOG 2	7	(4%)
Histology	Squamous cell carcinoma	86	(55%)
	Adenocarcinoma	52	(33%)
	Large cell carcinoma	2	(1%)
	NSCLC, NOS	17	(11%)
T stage	1	18	(11%)
	2	61	(39%)
	3	34	(22%)
	4	44	(28%)
N stage	0	3	(2%)
	1	3	(2%)
	2	75	(48%)
	3	76	(48%)
Stage	IIIA	49	(31%)
	IIIB	108	(69%)

*Abbreviations:* *ECOG* Eastern Cooperative Oncology Group, *NSCLC* Non-small-cell lung cancer, *NOS* Not otherwise specified.

### Radiotherapy

Ninety percent of patients (n = 142) were treated with three-dimensional conformal RT (3D-CRT). The remainder received intensity-modified radiotherapy (IMRT, n = 3) or helical tomotherapy (n = 12). All treatments were based on CT planning. The CT simulation was performed with GE LightSpeed RT (GE Healthcare, Milwaukee, WI, USA) or Picker CT-Simulator UltraZ (Philips Medical System, Best, The Netherlands), and each scan slice had 3–5 mm thickness. Patients were required to have shallow respiration, and scanned from lower neck to upper abdomen over 10 respiratory phases. The target volumes were defined as follows: GTV, primary tumor(s) and involved lymph node(s); clinical target volume (CTV), GTV +1 cm for microscopic tumor extension; planning target volume (PTV), CTV +5-15 mm margin. A minimum of 3–6 coplanar isocentric fields were designed for 3D-CRT and IMRT with Pinnacle radiotherapy treatment planning (RTP) systems (Philips Radiation Oncology Systems, Milpitas, CA, USA) or the Eclipse RTP system, version 8.3 (Varian Medical System Inc., Palo Alto, CA, USA). The Hi-Art Helical TomoTherapy RTP system, version 3.0 (TomoTherapy Inc., Madison, WI, USA) was used for helical tomotherapy planning. The median daily dose and total dose were 2 Gy (range, 1.8–2.4 Gy) and 63 Gy (range, 59.4–74 Gy), respectively. The 2 Gy equivalent total target doses were estimated as a median of 66.1 Gy (range, 57.3–73.8 Gy), with the assumptions of an α/β ratio as 10 Gy, effective doubling time as 5 days, and kick-off time of accelerated repopulation as 14 days. The details of RT are provided in Table 2.

**Table 2:** **Treatment characteristics**

**Characteristic**	**n**	**(%)**
Radiotherapy		
3D-CRT	142	(90)
Median 63 Gy		
(59.5–74 Gy/25–37 fractions)		
Tomotherapy	12	(8)
Median 66 Gy		
(66–70.4 Gy/27–35 fractions)		
IMRT	3	(2)
60 Gy		
(60 Gy/25–30 fractions)		
Chemotherapy		
Cisplatin, Etoposide	54	(34)
Carboplatin, Paclitaxel	50	(32)
Cisplatin, Paclitaxel	36	(23)
Cisplatin, Irinotecan	17	(11)

*Abbreviations:* *3D-CRT* Three-dimensional radiotherapy, *IMRT* Intensity-modulated radiotherapy.

### Chemotherapy

Chemotherapy regimens administered during RT were as follows (Table 2). Cisplatin (60 mg/m^2^ on days 1, 8, 29, and 36) and etoposide (100 mg/m^2^ on days 1–5 and 29–33) in 54 patients (34%), weekly carboplatin (area under the curve = 2, intravenously over 30 min) and paclitaxel (50 mg/m^2^ over 1 h) in 50 patients (32%), weekly cisplatin (20 mg/m^2^ on days 1, 8, 29, and 36) plus paclitaxel (50 mg/m^2^ over 1 h) in 36 patients (23%), and weekly cisplatin (30 mg/m^2^ on days 1, 8, 29, and 36) and irrinotecan (60 mg/m^2^) in 17 patients (11%).

### Follow-up

Patients were regularly followed after the completion of CCRT at 1 month, every 3 months for 2 years, and every 6 months thereafter. Follow-up examination routinely included chest radiographs and chest CT. Generally, the follow-up CT was scanned in a breath-holding period with a less than 3 mm of thickness. Our instituional policy has been to check short-term follow-up CT in 1 month after RT to evaluate response before the occurrence of radiation pneumonitis, because early radiation pneumonitis usually gets evident on imaging after a few months of RT completion [14].

Locoregional failure was defined as recurrence in the irradiated lung or in the regional lymph nodes. Failure in any other site was noted as distant metastasis. The Radiation Therapy Oncology Group (RTOG) and European Organization for Research and Treatment of Cancer (EORTC) radiation morbidity criteria were used to grade acute and late toxicities [15].

### Volumetric parameters

GTV was delineated at planning CT just before CCRT (GTV_pre_) and at the follow-up CT 1 month after CCRT (GTV_post_). To minimize confusion between disease and pneumonitis, the follow-up CT 1 month after CCRT was selected to delineate GTV_post_. We registered all image data in the Pinnacle RTP system for delineation. The volume reduction ratio of GTV (VRR), implying the short-term response rate, was defined as % (GTV_post_ – GTV_pre_)/GTV_pre_.

For the accuracy and consistency, GTVs were delineated by a single physician and reviewed by two additional physicians. GTV_pre_ were defined as primary and involved lymph nodes in the original RT planning. The criteria of involved lymph nodes were as follows: pathologic confirmation or diameter of > 10 mm. When 18 F-fluorodeoxyglucose positron emission tomography (FDG-PET) was scanned, standardized uptake values were referred to delineate GTV_pre_. For example, small lymph nodes might be considered to be involved if showed high uptake. To prevent over-estimation of GTV_pre_, CT images in a single respiratory phase were used rather than maximum intensity projection. We traced GTV_pre_ in the follow-up CT and delineated as GTV_post_. In case of atelectasis, tumor was distinguished from collapsed lung tissues by using serially scanned CT images or FDG-PET.

### Statistics

Kaplan-Meier analysis was used to estimate survival rate, set the primary endpoint overall survival (OS), the secondary progression-free survival (PFS) and locoregional progression-free survival (LRPFS). To compare survival differences, the log-rank test and Cox’s regression model were used for categorical and continuous variables, respectively. The observed differences were regarded statistically significant if the *p* value was < 0.05, and as non-significant trends or borderline significance if the *p* value was < 0.1. Multivariate analysis was performed by incorporating variables shown to be significant or borderline significant in univariate analysis, in addition to well-known prognostic factors. The Cox proportional-hazards model was used to calculate hazard ratios (HR) in multivariate analysis. All statistical analyses were performed using SPSS version 18.0 (SPSS Inc., Chicago, IL).

Volumetric parameters were analyzed as both continuous and categorical variables. Hypothetical candidate cut-off values of GTV_pre_, GTV_post_, and VRR with optimal ranges were tested by the statistical method proposed by Contal and O’Quigley [16], which is based on the log-rank test. Thereafter, both the receiver operating characteristic (ROC) curve and a maximal χ^2^ method were used to identify the best cut-off values of volume parameters using the software package R 2.13.0 (R Development Core Team, Vienna, Austria, http://www.R-project.org). A total of 20 categorical values, from 10 to 200 cm^3^ for GTV_pre_ and GTV_post_ were tested as candidates for the best cut-off value by dividing significantly different OS groups. For VRR, nine candidates of 10% intervals (from 10–90%) were hypothesized.

## Results

### Patients’ characteristics

Of the 157 patients, 79 patients (50%) had T1 and T2 tumors, and 78 patients (50%) had T3 and T4 tumors. Eighty-six patients (55%) had squamous cell carcinoma (SqCC), and 52 patients (33%) had adenocarcinoma (ADC). At the time of diagnosis, 150 patients (96%) had an Eastern Cooperative Oncology Group performance status of 0 to 1. Patients’ characteristics are provided in Table 1.

### Survival outcomes

**Figure 1:**
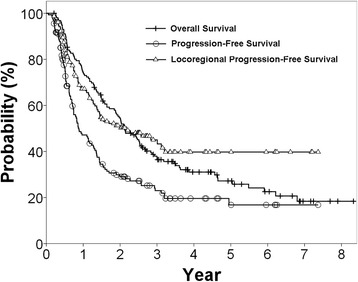
**Kaplan-Meier survival curves for study patients.**

The median follow-up time for all patients was 24.4 months (range, 2.5–99.9 months) and for surviving patients was 52.7 months (range, 27.4–99.9 months). The median OS was 25.5 months and the estimated 3-year OS rate was 36.4%. The 3-year PFS was 23% (median, 10.6 months) and the 3-year LRPFS was 45.0% (median, 25.7 months), as shown in Figure 1.

### Univariate analysis of cut-off values for volumetric parameters related to survival outcomes

The median values of GTV_pre_, GTV_post_, and VRR were 96.7 cm^3^ (range, 9.7–1169.8), 25.7 cm^3^ (range, 1.87–249.1), and 70.9% (range, 1.7–96.9), respectively. The distributions of GTVs were normal on a natural logarithmic scale, except VRR (Additional file 1: Figure S1). Through statistical processing of the ROC curve and the maximal χ^2^ test described above, possible cut-off values were estimated as 50 or 60 cm^3^ for GTV_pre_, 10 or 20 cm^3^ for GTV_post_, and 50 or 60% for VRR. The ideal cut-off value was selected from the maximum difference between the two groups as 50 cm^3^ for GTV_pre_, 20 cm^3^ for GTV_post_, and 50% for VRR.

The results of univariate analyses of clinical and volumetric factors with OS, PFS and LRPFS are shown in Table 3. Females had better OS outcomes than males (*p =* 0.017) and patients with stage IIIA disease had better PFS than patients with stage IIIB (*p* = 0.041). The LRPFS of patients with squamous histology (*p =* 0.048) and T3-4 (*p =* 0.034) was inferior to that of patients with non-squamous histology and T1-2, respectively.

**Table 3:** **Univariate analysis of volumetric parameter cut-off values compared to survival outcomes**

**Variable** ^*^		***n***	**3Y LRPFS (%)**	***p*** ^**†**^	**3Y PFS (%)**	***p*** ^**†**^	**3Y OS (%)**	***p*** ^**†**^
Sex	Male	137	41.6	0.251	22.9	0.589	36.6	0.017
	Female	20	63.3		23.8		55.0	
Age	≤ 60 y	65	41.3	0.563	20.5	0.444	35.5	0.307
	> 60 y	92	47.9		25.0		37.1	
ECOG	0	39	32.6	0.611	15.0	0.318	34.4	0.721
	1-2	118	47.8		25.5		36.8	
Histology	SqCC	86	36.0	0.048	24.0	0.517	32.0	0.151
	Others	71	56.3		19.2		41.8	
	ADC	52	52.5	0.110	19.2	0.596	45.0	0.335
	Others	105	40.5		24.9		34.6	
T stage	1-2	79	56.4	0.034	25.2	0.281	43.8	0.106
	3-4	78	33.8		20.7		28.7	
N stage	0-2	81	49.4	0.448	29.2	0.108	37.0	0.686
	3	76	39.5		17.0		35.6	
Stage	IIIA	49	56.5	0.084	33.1	0.041	42.0	0.705
	IIIB	108	39.8		18.6		33.7	
GTV_pre_	(Continuous)	(95% CI)	(1.001)	(0.330)	(1.003)	(<0.001)	(1.001)	(0.019)
		(HR)	(0.999–1.002)		(1.002–1.004)		(1.000–1.002)	
	≤ 50 cm^3^	33	75.0	0.001	42.5	0.001	65.7	< 0.001
	> 50 cm^3^	124	34.8		17.1		28.4	
GTV_post_	(Continuous)	(95% CI)	(1.004)	(0.189)	(1.015)	(<0.001)	(1.006)	(0.008)
		(HR)	(0.998–1.010)		(1.008–1.021)		(1.002–1.011)	
	≤ 20 cm^3^	68	53.3	0.165	32.4	0.004	43.2	0.015
	> 20 cm^3^	89	36.7		15.5		31.0	
VRR	(Continuous)	(95% CI)	(1.011)	(0.126)	(1.008)	(0.139)	(1.013)	(0.028)
		(HR)	(0.997–1.025)		(0.997–1.020)		(1.001–1.025)	
	≤ 50%	21	68.2	0.042	39.6	0.054	64.6	0.004
	>50%	136	40.6		20.2		32.2	

*Abbreviations:* *ADC* Adenocarcinoma, *CI* Confidence interval, *ECOG* Eastern Cooperative Oncology Group, *GTV*_*pre*_ Initial gross tumor volume, *GTV*_*post*_ Follow-up gross tumor volume, *HR* Harzard ratio, *LRPFS* locoregional progression-free survival rate, *OS* Overall survival rate, *SqCC* Squamous cell carcinoma, *VRR* Reduction ratio of gross tumor volume.

^*^All variables are categorical, unless being noted as “continuous”.

^†^The log-rank test and the Cox proportional hazards model were used for categorical and continuous variables, respectively.

**Figure 2:**
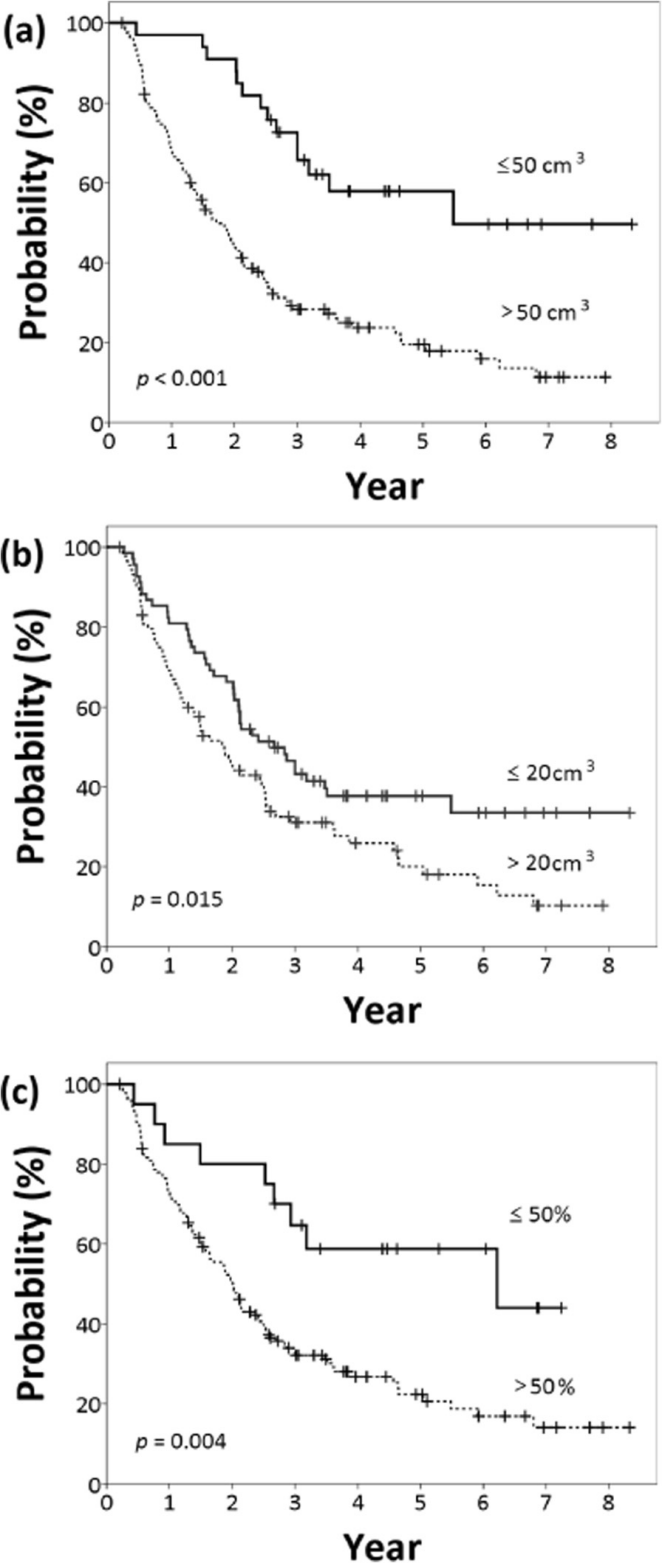
**Comparison of overall survival curves according to gross tumor volume: (a) pre-treatment, (b) post-treatment, (c) volume reduction ratio.**

Patients with smaller GTV_pre_ had significantly better OS (*p <* 0.001), PFS (*p =* 0.001) and LRPFS (*p =* 0.001). Smaller GTV_post_ was also indicative of better OS (*p =* 0.015) and PFS (*p =* 0.004). Patients with higher VRR had worse OS (*p* = 0.004). The OS curves related to volumetric parameters are shown in Figure 2.

### Multivariate analysis of cut-off values for volumetric parameters related to survival outcomes

Based on the results of the univariate analyses, we performed a multivariate analysis with a stepwise backward selection procedure, incorporating clinical variables and categorical volumetric parameters (Table 4). Smaller GTV_pre_ was found to be the only significant prognostic factor of better LRPFS (HR = 2.926; *p* = 0.002), PFS (HR = 2.001; *p* = 0.013), and OS (HR = 2.763; *p* = 0.001), while a smaller GTV_post_ tended to be a prognostic factor of better PFS (HR = 1.467; *p* = 0.086). Higher VRR was marginally significant for poorer OS (HR = 1.895; *p* = 0.075). Squamous histology also tended to predict a poorer PFS (HR = 1.455; *p* = 0.061).

**Table 4:** **Multivariate analysis of volumetric parameter cut-off values compared to survival outcomes**

	**LRPFS**		**PFS**		**OS**	
	**HR (95% CI)**	***p***	**HR (95% CI)**	***p***	**HR (95% CI)**	***p***
Sex (Male vs. Female)		NS		NS		NS
Histology (SqCC vs. Others)		NS	1.455 (0.983–2.155)	0.061		NS
T stage (T1-T2 vs. T3-T4)		NS		NS		NS
N stage (N0-N2 vs. N3)		NS		NS		NS
Stage (IIIA vs. IIIB)		NS		NS		NS
GTV_pre_ (≤ 50 cm^3^ vs. > 50 cm^3^)	2.926 (1.495–5.726)	0.002	2.001 (1.159–3.454)	0.013	2.763 (1.552–4.919)	0.001
GTV_post_ (≤ 20 cm^3^ vs. > 20 cm^3^)		NS	1.467 (0.947–2.273)	0.086		NS
VRR (≤ 50% vs. > 50%)		NS		NS	1.895 (0.937–3.832)	0.075

*Abbreviations:* *CI* Confidence interval, *GTV*_*pre*_ Initial gross tumor volume CT, *GTV*_*post*_ follow-up gross tumor volume, *HR* Hazard ratio, *LRPFS* Locoregional progression-free survival rate, *NS* No significance, *OS* Overall survival rate, *PFS* Progression-free survival rate, *SqCC* Squamous cell carcinoma, *VRR* Reduction ratio of gross tumor volume.

### Toxicity

During treatment, grade III or IV hematologic adverse events were noted in 26 patients; no life-threatening events were recorded. Radiation esophagitis developed in 146 patients (42% with grade I, 46% grade 2, and 12% grade 3). A total of 143 patients experienced radiation pneumonitis (54% with grade I, 38% grade 2, 4% grade 3 and 3% grade IV). In five patients, treatment-related pneumonitis was aggravated; these individuals ultimately succumbed 6 months after treatment completion.

## Discussion

Several volumetric studies have been conducted to evaluate initial GTV as a prognostic factor in patients with NSCLC who underwent definitive RT [6-12]. However, the prognostic role of residual tumor volume or tumor response after treatment was assessed only in trials of induction therapy for NSCLC [8,17,18], not definitive CCRT. To our knowledge, the present study is the first to evaluate the prognostic role of volumetric parameters and include GTV_pre_, GTV_post_, and VRR in patients with NSCLC who underwent definitive CCRT.

In our study, GTV_pre_ was an independent prognostic factor of survival in patients with CCRT for locally advanced NSCLC, which is consistent with previous reports. In a secondary analysis of the RTOG 93–11 phase I-II radiation dose-escalation study by Werner-Wasik et al. [7], patients with larger GTV, defined as the sum of the volumes of the primary tumor and involved lymph nodes, had a shorter median survival time and PFS than patients with smaller GTV. Basaki et al. [10] also evaluated the impact of tumor volume on OS in stage III NSCLC patients (n = 71) treated with definitive RT using sequential or concurrent chemotherapy, and found that both the total tumor volume and the primary tumor volume were significant, while the nodal volume was not. In a study by Alexander et al. [11], both tumor and nodal volume were associated with OS and local control, but not with distant metastasis in patients with stage III NSCLC (n = 107) treated by chemoradiotherapy with or without surgery.

Current evidence of the prognostic significance of GTV_pre_ indicates that the larger the entire tumor burden, the more difficult it is to achieve a log cell kill due to the quantity of cancer cells to be destroyed and the higher proportion of radio-resistant area. Several studies segregated nodal volume from the entire tumor volume with the hypothesis that volumes of primary tumor and involved lymph nodes might represent local and systemic NSCLC disease, respectively. Nodal volume might provide additional information to the N stage; however, the prognostic role of nodal volume is controversial [4,10,11]. In the current study, we did not perform subgroup analysis of nodal volume in GTV_pre_, because segregated nodal volume delineation was difficult in a proportion of patients due to lymph nodes conglomerated with primary tumor. To evaluate the prognostic role of primary tumor and nodal volumes, fractional delineation using an advanced imaging technique, such as FDG-PET, might be necessary [19,20].

Although Yamane et al. [21] suggested the area of residual tumor after neoadjuvant therapy as a prognostic factor, GTV_post_ in our study was not significantly associated with OS in multivariate analysis, despite a trend toward improved PFS. The short-term response rate has frequently been used as a surrogate marker for survival in novel oncology agent clinical trials. However, there is little evidence of the influence of induction chemotherapy response on survival [17,22,23]. In contrast, the short-term response rate, evaluated 4–6 weeks after induction CCRT followed by surgical resection, has been reported as 35–66%, suggesting that the complete pathological response is a significant predictor of prognosis [24-27]. At this point, it is unclear whether a better short-term response after definitive CCRT leads to improved prognosis. However, it is known that if surgery is technically feasible, it can benefit a small proportion of patients.

Based on this, it is notable that a VRR > 50% after definitive CCRT had a negative impact on OS in univariate analysis and a similar trend in multivariate analysis. Since the cut-off point of VRR in our study was 50%, which is also the cut-off value dividing partial response and stable disease in the conventional response criteria of the World Health Organization [28] that measures volume bi-dimensionally, better-responding patients after short-term follow up were not guaranteed to have an improved outcome without surgery in our study. Our interesting finding may be reasonable, considering GTV_post_ contains viable residual tumor as well as radiation injury. We used CT alone to delineate GTV_post_, however, CT has a major restriction to distinguish persistent or recurrent tumor from treatment-induced changes early after the completion of RT [14]. It appears that the addition of FDG-PET to CT is superior to CT alone in the evaluation of persistent or recurrent tumor in patients undergoing RT for NSCLC, as well as in the delineation of primary lesion [29-31]. Therefore, our unexpected finding of VRR and GTV_post_ should be interpreted that response evaluation at certain time point may be ambiguous with CT alone, so functional imaging technique, such as FDG-PET, is a reasonable complement for early response assessment.

In NSCLC, treatment sensitivity or failure patterns differ between squamous cell carcinoma (SqCC) and adenocarcinoma (ADC), with either surgery or RT [32-34]. Ishikawa et al. [33] analyzed the recurrence pattern and survival of patients with stage I NSCLC treated with definitive RT. There was no significant difference in survival; however, the 5-year primary control rate of SqCC was worse than that of ADC (62 *vs.* 88%, *p* = 0.03), and the 5-year metastasis-free survival rate of SqCC was better than that of ADC (88.2 *vs.* 53.0%, *p* = 0.005). In the current study, patients with SqCC had worse LRPFS (*p =* 0.048), which was not significant in multivariate analysis.

Our findings have clinical implications, since we analyzed possible associations between all volumetric parameters incorporating GTV_pre_, GTV_post_ and the VRR in definitive CCRT with survival outcomes. Furthermore, to minimize inter-observer variation attributed to variability in target delineation, GTV_pre_ and GTV_post_ were defined by a single physician, and consistencies of contours were supervised by two physicians. Target delineation method of this study, as described above, could contribute to decrease inter-observer variations, also. Despite this, there are some limitations. For example, the patient population was not homogenous in terms of RT dose fractionation, planning technique, and chemotherapy regimens since the data were not extracted from prospective clinical trials. It was also difficult to evaluate the effect of salvage treatment on the outcome of patients with disease progression after definitive CCRT. Additionally, this retrospective study did not use FDG-PET/CT for routine surveillance, which may be helpful to assess early response of tumor.

In conclusion, smaller GTV_pre_ was an independent prognostic factor of better prognosis in patients with stage III NSCLC treated with definitive CCRT. GTV_post_ did not have association with survival on the multivariate analysis. Notably, improved outcome was not correlated with > 50% VRR after short-term follow-up with CT alone. Our findings of GTV_post_ and VRR, in NSCLC patients having definitive CCRT, should be clarified with a prospective study of short-term response assessment using functional imaging technique, such as FDG-PET/CT.

## Conclusions

We confirmed that smaller GTV_pre_ was associated with better survival. Interestingly, GTV_post_ did not have an association with survival on the multivariate analysis; higher VRR had a trend of poorer survival. GTV_pre_ is a significant prognostic factor for survival. Short-term follow-up using CT alone may not be enough for response assessment in NSCLC patents having definitive CCRT. A prospective study using functional imaging modality is needed to evaluate the prognostic role of early response assessment in NSCLC patients having definitive CCRT.

## Additional file

Additional file 1: Figure S1. Gross tumor volume has a normal distribution on natural logarithmic scale: (a) pre-treatment, (b) post-treatment. Volume reduction ratio is not normally distributed (c).
