# Supplementary figures and images for: The effect of tumor volume and its change on survival in stage III non-small cell lung cancer treated with definitive concurrent chemoradiotherapy
Source: Radiat Oncol. 2014 Dec 13;9:283. doi: 10.1186/s13014-014-0283-6 (PMC4268851; doi:10.1186/s13014-014-0283-6)

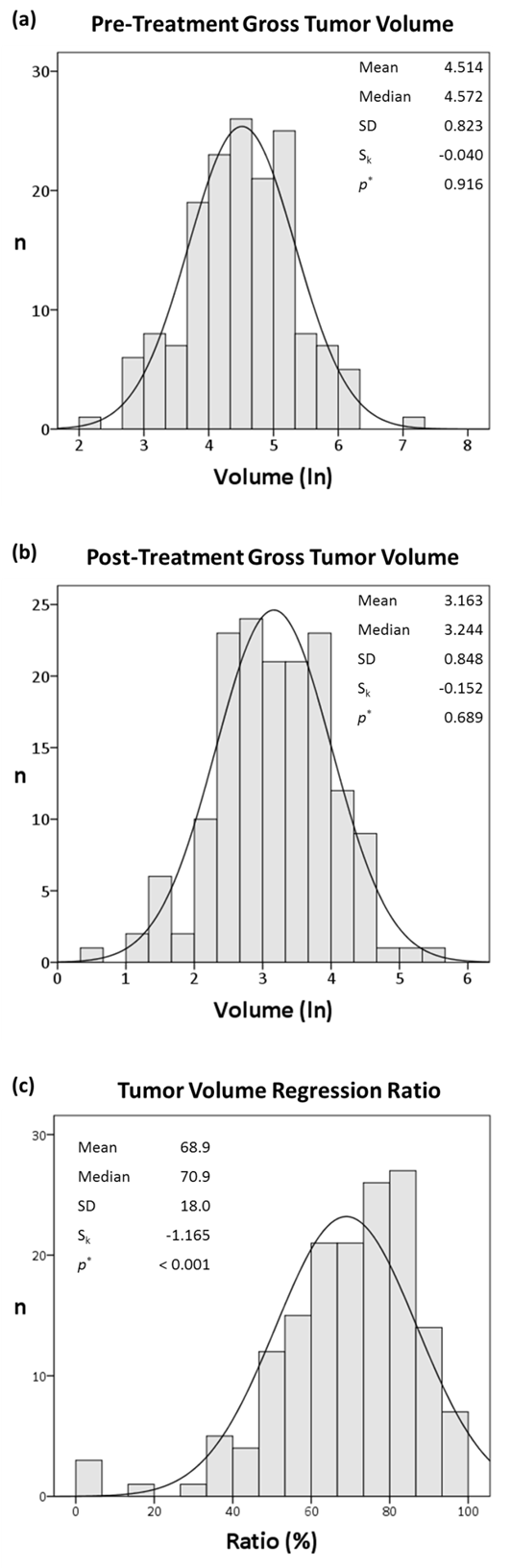

Supplement: Additional file 1: Figure S1. — Gross tumor volume has a normal distribution on natural logarithmic scale: (a) pre-treatment, (b) post-treatment. Volume reduction ratio is not normally distributed (c). [file 13014_2014_283_MOESM1_ESM.tiff]
